# Supplementary material for: Long-Term Usage of Proton Pump Inhibitors Associated with Prognosis in Patients with Colorectal Cancer
Source: Cancers (Basel). 2023 Nov 6;15(21):5304. doi: 10.3390/cancers15215304 (PMC10648487; doi:10.3390/cancers15215304)
Supplement: Supplementary file 1 [file cancers-15-05304-s001.zip › cancers-2675371-supplementary.pdf]

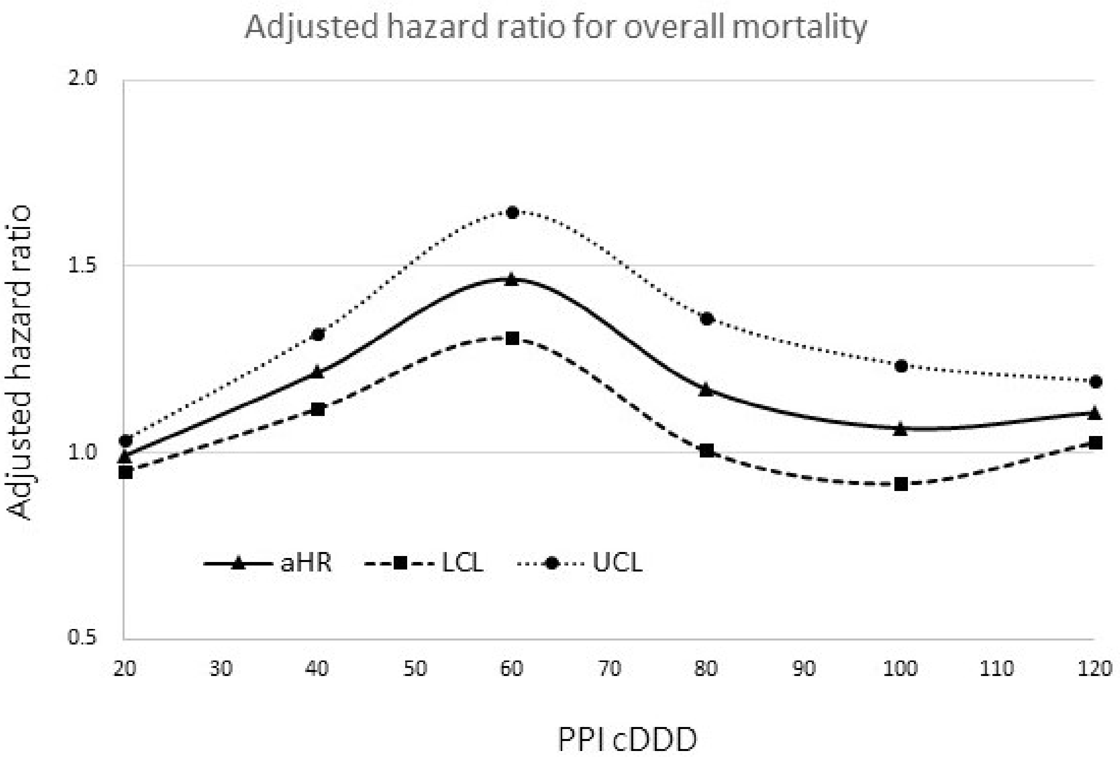

Figure S1: The Dose-Response of Proton Pump Inhibitors in Overall Mortality.

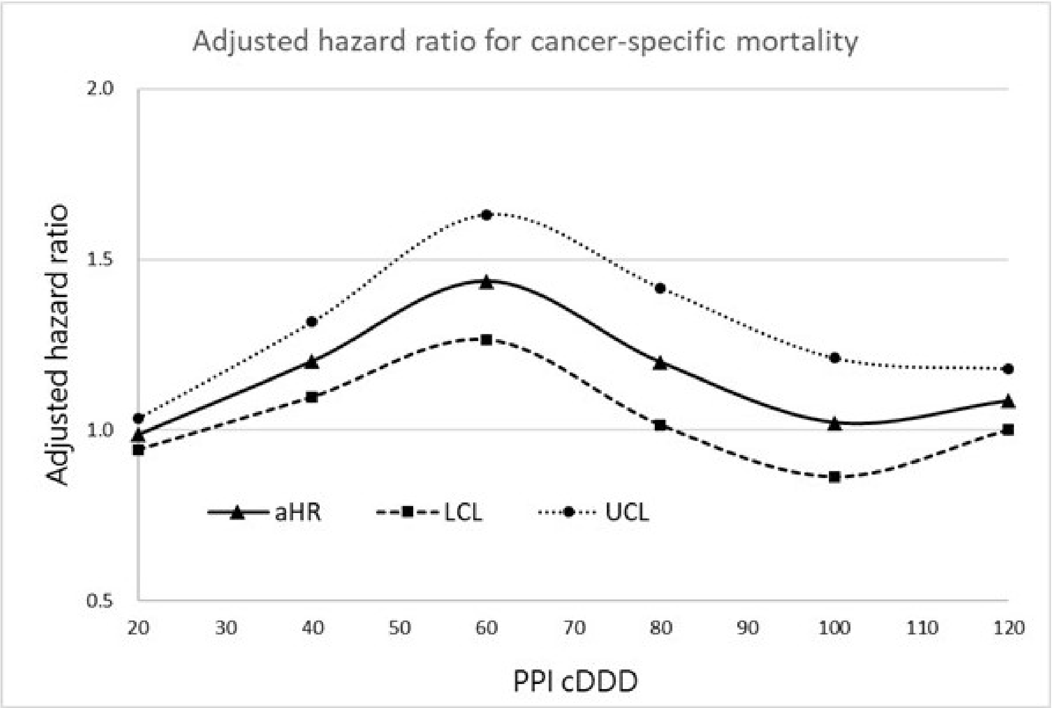

Figure S2: The Dose-Response of Proton Pump Inhibitors in Cancer-Specific Mortality.

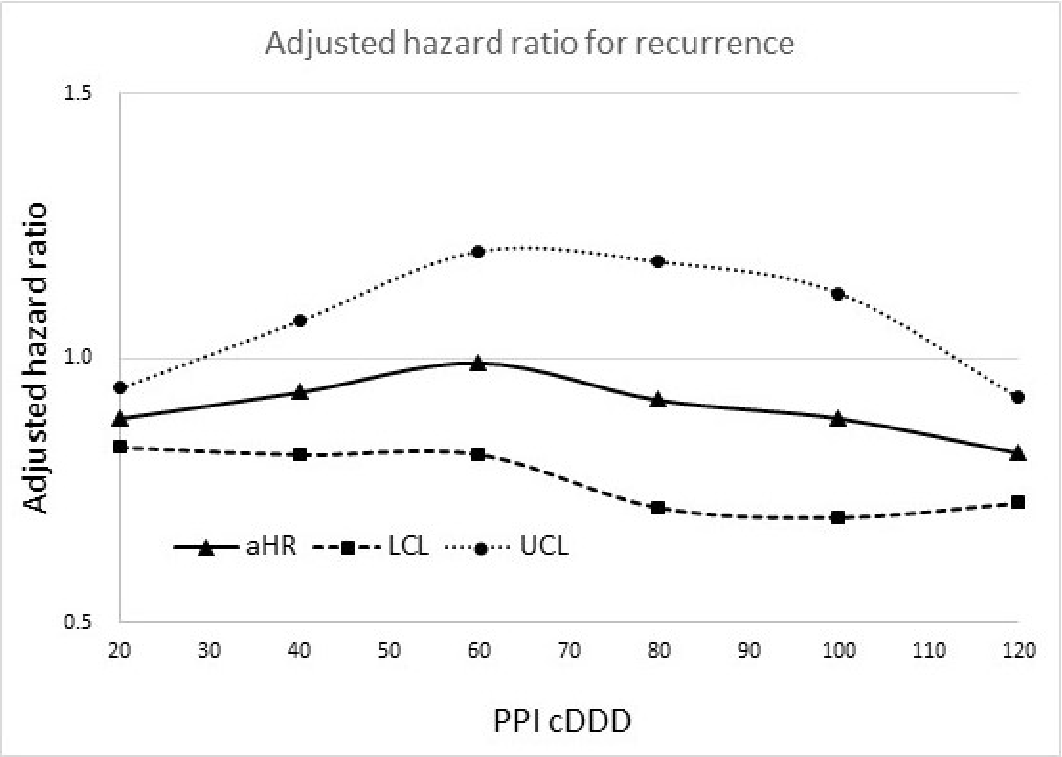

Figure S3: The Dose-Response of Proton Pump Inhibitors in Cancer Recurrence.

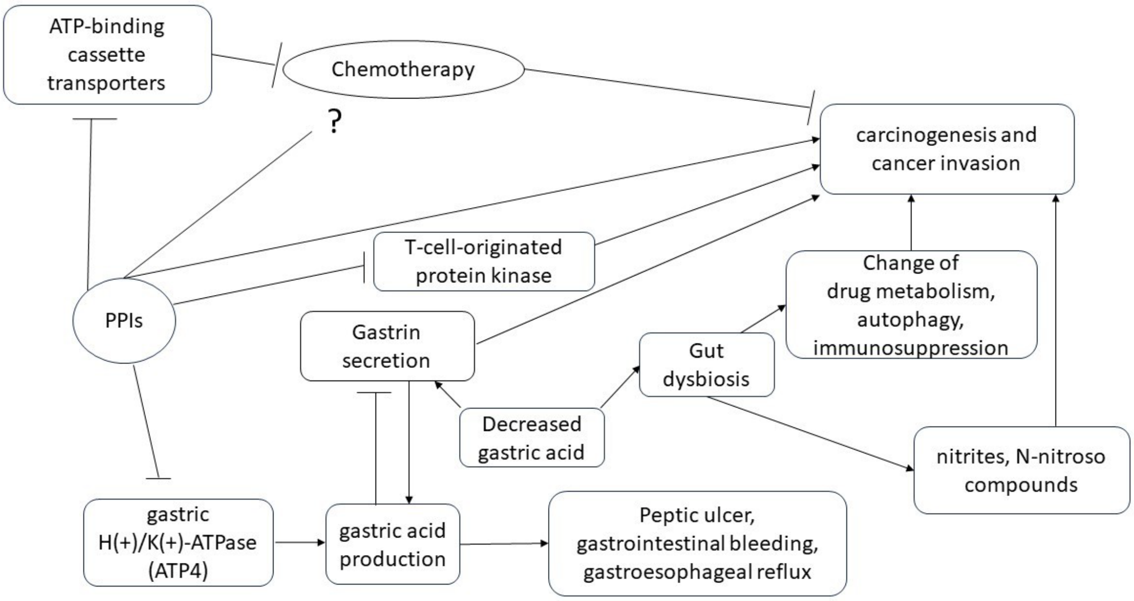

Figure S4: Schematic Representation of PPI Effects in Colorectal Cancer.
